# Recent advances in understanding and managing Lichen Sclerosus

**DOI:** 10.12688/f1000research.21529.1

**Published:** 2020-05-15

**Authors:** Rachel Kwok, Taimur T. Shah, Suks Minhas

**Affiliations:** 1 Division of Surgery, Department of Surgery and Cancer, Faculty of Medicine, Imperial College London, London, UK; 2 Department of Urology, Charing Cross Hospital, Imperial College Healthcare NHS Trust, London, UK

**Keywords:** Lichen Sclerosus, Balanitis Xerotica Obliterans (BXO), Penile Cancer

## Abstract

Lichen sclerosus (LS), or balanitis xerotica obliterans as it was previously known, is a chronic inflammatory lymphocyte-mediated scarring dermatosis that often affects the preputial skin and glans, leading to phimosis and urethral strictures if left untreated. We present a narrative review of the literature assessing its aetiology and pathogenesis and discuss its links to penile cancer and its medical and surgical management. Possible hypotheses for the development of LS include chronic exposure to trapped urine, leading to changes in the epithelial structure. This is supported by the fact that circumcision is often curative in the early stages of the disease. Although circumcision can be curative, the use of topical steroids is typically the first-line treatment and may preserve the foreskin and forgo the need for circumcision altogether. Patients should be made aware of a possible association with penile cancer. Although the majority of cases can be treated by medical therapy and circumcision, a significant number of patients may also require penile reconstructive procedures.

## Introduction

Lichen sclerosus (LS) is a chronic inflammatory lymphocyte-mediated scarring dermatosis that presents mainly in the anogenital area of both sexes ^1, 2^. Although LS presents more frequently in women, this review focuses only on LS in men, which was formerly known as balanitis xerotica obliterans ^3^.

## Aetio-pathogenesis and pathology

The aetiology of LS in men remains unclear, although a number of theories have been proposed to explain its aetio-pathogenesis ( Table 1). There is little evidence of a genetic predisposition for male LS, although this has been implicated in women ^4^. Associations between male LS and certain human leucocyte antigens (HLAs) have also been found, but similar HLAs also appeared to be protective in female vulvar LS ^4, 5^. Therefore, it is inconclusive whether genetic factors are involved in an individual’s risk in developing LS.

**Table 1.** Summary of potential factors involved in aetio-pathogenesis of male lichen sclerosus.

Potential aetiological factor		Evidence
Genetic ^4^	Family history	Low level
	Human leucocyte antigen (HLA) ^5^	Increased frequencies of HLA-DR11, -DR12 and -DQ7
	Autoimmunity	Association with atopic eczema in boys No association with selected filaggrin loss-of-function mutations (R501X, 2282del4, R2447X and S3247X) ^9^
Autoimmunity		No specific gene expression association observed Gene expression interpreted as non-specific inflammatory tissue response Only 7% of patients (n = 23) in a case series had associated autoimmune conditions (alopecia areata, vitiligo, thyroid disease and ulcerative colitis) ^11^ Fewer patients with male lichen sclerosus (LS) had antinuclear antibodies or autoimmune diseases than general population ^15, 16^ Autoantibodies to extracellular matrix protein 1 have been observed but may be an epiphenomenon ^4^.
Infection	Human papilloma virus (HPV)	No gene expression association ^12^ Transcriptosome of male LS unrelated to HPV Immunostaining observed association of HPV16/18 and LS in 6/18 cases ^4^ Median HPV prevalence of 29% in male LS found in a systematic review ^12^
	Others	*Fusobacterium spp*. present in more than 70% of male genital LS ^13^ No association with *Borrelia burgdorferi* ^17^
Exposure to urine		Associated with conditions with increased occlusion and urine contact ^8^ Low incidence in circumcised men ^2^

Over the last decade, it has been suggested that the chronic occluded exposure of urine (or a urine substituent) to susceptible epithelium could be involved in the aetio-pathogenesis of male genital LS ^6^. LS rarely occurs in men with a history of neonatal circumcision, suggesting that the foreskin plays an important role in its pathogenesis ^2^. However, circumcision may lead to scarring that predisposes patients to an acquired adult buried penis and also the development of LS possibly from chronic urine exposure ^7^. Generally, the area between the foreskin and glans penis is an intertriginous site, which may trap moisture ^2^ or allow the development of dermatoses. LS is associated with hypospadias, urethrostomies, urostomies and post-micturition micro-incontinence, suggesting that urine contact, when urinary occlusion is present, supports the development of LS ^8^. Conversely, although a significant filaggrin loss-of-function mutation-related skin-barrier defect is often observed in patients with atopic eczema, such a defect could not be detected in men with genital LS ^9^. Additionally, mass resonance spectroscopy has been unable to elucidate the culpable constituent of urine ^8, 10^. These findings do not support the theory that urine is implicated in the aetio-pathogenesis of male genital LS. Further studies – prospective and controlled studies – are needed to determine the role and mechanism of urine in irritating male genital epithelial surfaces.

The gene expression profiling of male genital LS shows no evidence of association with either autoimmune diseases or the human papilloma virus (HPV), suggesting that these are not relevant in the pathogenesis of male LS ^2, 11^. However, a systematic review reported that HPV was identified in 29% of male LS cases ^12^; ongoing debate about the role that HPV plays in the aetiology of LS therefore persists. More recently, it has been observed that the *Fusobacterium spp.* is present in over 70% of patients with male genital LS ^13^, and circumcision was found to decrease the abundance of this pathogen, which may explain its curative effect ^14^. Further studies should be performed to elucidate the role of *Fusobacterium spp*. and other infective or microbiomal agents in the aetiology of LS. Other studied potential autoimmune or infectious causes are described in Table 1.

Additionally, it has been found that male LS is associated with an increased body mass index, coronary artery disease, diabetes mellitus and smoking. This leads to the hypothesis that systemic metabolic factors or inflammation affecting microvascular health contributes to both the development and chronicity of this condition ^18, 19^. Vascular compromise is known to affect the most distal end of the penis and urethra, which may explain the distal-to-proximal progression of LS in the formation of urethral strictures ^18^. The aetiology of male LS is therefore likely to be multi-factorial. Exploratory work by Levy *et al*. revealed that, in men, LS-associated urethral strictures had a lower Ki-67 mitotic index but raised levels of vascular endothelial growth factor ^20^. The authors also found that LS strictures were longer with higher levels of inflammatory markers than non-LS strictures with raised markers for HPV, varicella zoster and Epstein–Barr virus, raising the possibility of an infectious cause ^21^. Ultimately, the aetiology of male LS is likely to be multi-factorial.

## Incidence and epidemiology

The estimated incidence of male LS has been reported as 0.07 to 0.3%, and a bimodal distribution with peak ages of presentation in young boys and adult men was reported ^2, 3, 22^. The prevalence is reported to be highest in men who are 61 years old or older ^2, 3^. The exact incidence may be higher because of the under-reporting by physicians unfamiliar with the condition, asymptomatic presentations, or patient fear or embarrassment ^5, 23^. Furthermore, without routine pathological evaluation of foreskin following circumcision, some LS diagnosis may have been missed.

The reported incidence of LS in foreskin samples obtained in boys under 18 years of age following circumcision and confirmed with histopathology was 35% ^24^. In men, 4 to 19% of foreskin biopsies following circumcision revealed histological findings of LS ^25^. However, it is important to note that these data cannot be compared directly as the indications for circumcision may have been different in each group.

## Association with penile cancer

Genital LS has been associated with penile squamous cell carcinoma (SCC) and the estimated lifetime risk is 4 to 5% ^2, 26^; 23 to 40% of penile carcinomas were associated with concurrent histological evidence of LS ^2^, and the time interval between LS diagnosis and the development of penile SCC was found to be 10 to 23 years ^26^. However, LS was not found to be associated with more aggressive histopathological features in penile SCC, including carcinoma *in situ* ^27^. A direct causal relationship between LS and penile SCC has not been found thus far and cannot be established from only observational studies; this is due to the inherent potential of observation bias because of the rarity of penile SCC, which has an incidence of 0.1 to 0.9 cases per 100,000 men in Europe, whereas LS is a relatively common condition ^27^. Primary penile melanoma has also been reported in patients with genital LS, although at present there is no evidence of an association between the two conditions ^28^. However, all patients with a diagnosis of LS should be advised of the association and should regularly perform self-examination and have regular long-term follow-up even if asymptomatic.

Penile SCC has many potential risk factors, including phimosis with chronic inflammation and HPV infection ^27^; the role of HPV in the aetio-pathogenesis of LS is still unclear, but HPV could be a common risk factor for both conditions, making a direct pathogenic link between LS and SCC difficult to establish. There are also two potential pathways for penile SCC; one is related to HPV and the other is related to chronic scarring dermatoses ^2^. Case series with polymerase chain reaction analysis for high-risk HPV presence provided discordant results ^27^ and do not explain the relationship between LS, penile SCC and HPV infection. Phimosis is another confounding potential risk factor for penile SCC since LS can also occur in phimosis ^29^. Whether LS is a premaglignant lesion remains unclear.

It has been suggested that factors such as chronic inflammation, tumour suppressor gene p53 mutations, and oxidative DNA damage lead to the malignant transformation of LS to penile SCC ^26^. This is supported by evidence (1) that the expression of tumour suppressors p16I ^INK4^ and p27 ^Kip1^ is downregulated in LS and (2) that a biomarker of oxidative stress, 8-hydroxy-deoxy-guanosine, increases as LS progresses into neoplasia, although this study ^30^ was undertaken in vulvar LS only.

## Presentation and clinical features

Male LS commonly presents as atrophic and white, hypertrophic and scaly, or violaceous plaques with telangiectasia and purpura ^4, 6^, often involving the glans penis, frenulum and prepuce but rarely the perianal area ^2^. A common presenting complaint is dyspareunia, difficulty with erections or sexual intercourse, due to discomfort or tearing of the foreskin ^2, 6^. Itch, blistering, bruising, bleeding, erosions or urinary symptoms are possible but less common in the male presentation of genital LS ^6^.

Additionally, phimosis may be caused by preputial scarring or the tightening of the foreskin ^2^, constrictive posthitis, which often is associated with ‘waisting’ of the distal penile shaft from a fibrotic band on retraction of the foreskin ^4, 6^. Other presentations may include adhesions, transcoronal or subcoronal, and frenulum scarring ^2^. Male LS is associated with adult buried penis, which sometimes may be a recurrence, arising from a previous circumcision where residual skin folds or a pseudo-foreskin remains because of obesity ^31^.

Isolated bulbar urethral strictures have also been found in men with LS; alternatively, peri-meatal disease can progress proximally to involve the fossa navicularis, causing pan-urethral stenosis which results in problematic voiding or in urinary retention and renal failure in more severe cases ^2, 23^. It has been suggested that 10% of all male urethral strictures could be caused by LS ^23^. Differential diagnoses of LS include infective balanitis, squamous neoplasia, plasma cell (Zoon’s) balanitis, mucosal or erosive lichen planus, and psoriasis ^23, 32^.

## Management

The main aims for the management of LS are to exclude malignant transformation, provide symptomatic relief, minimise urinary or sexual morbidity, and mitigate malignant transformation and preserve foreskin when feasible ^4, 6, 33^. All management strategies should be combined with conservative measures, such as using an emollient as a soap substitute and skin barrier ^2^, minimising contact with irritant factors (for example, urine after micturition) ^33^. Short, trimmed pubic hair is also recommended to minimise penile irritation ^33^.

It is unproven whether early corticosteroid or surgical treatment of LS mitigates the risk of malignant transformation, although a recent retrospective review of 301 patients found no progression to penile SCC in their cohort ^33^. A longitudinal prospective cohort study also found that, in women with vulvar LS, a long-term corticosteroid regimen reduced scarring and the risk of vulvar SCC ^34^. Therefore, follow-up monitoring and potential treatment remain essential ^6^.

## Medical management

The first-line management for LS in men and boys consists of potent topical corticosteroids, such as clobetasol propionate 0.05% ointment once or twice daily for 1 to 3 months – in accordance with the British Association of Dermatology (BAD) ^2^ and European ^35^ guidelines – with a repeat course in the case of a relapse. Corticosteroid treatment should be combined with weight loss if the patient is obese since a buried penis can impair treatment efficacy ^2^. Topical corticosteroid treatment relieves clinical symptoms and may also resolve phimosis ^32^; it has been observed that 50 to 60% of men are cured by short-term topical treatment and have minimal side effects ^33^. However, whether a long-term maintenance dose of topical corticosteroids should be recommended after clinical resolution remains controversial ^23, 32^. An individualised corticosteroid regimen, following disease remission, was observed to improve patient outcomes in vulvar LS ^34^.

A recent case report suggests that long-term topical corticosteroid use is safe and could prevent LS complications such as irreversible phimosis and malignancy, although side effects of such treatment, such as epidermal atrophy and herpes simplex virus infections, are difficult to elucidate as they can occur independently of corticosteroid use ^36^.

Calcineurin inhibitors (for example, tacrolimus and pimecrolimus) provide an effective off-label medical treatment option for male genital LS, although their response rates are lower than those of topical corticosteroids ^23^. Therefore, BAD guidelines suggest that any patient who fails to respond to 1 to 3 months of topical corticosteroid treatment should be referred for further evaluation and possibly to a urologist for circumcision ^2^.

## Surgical management

The mainstay of surgical management of male LS is circumcision, which typically is recommended after the failure of topical corticosteroid treatment, especially in early-stage uncomplicated cases ^1^. This has been found to cure more than 75% of patients ^33^. It has been hypothesised that circumcision allows the glans to fully keratinise ^6^ and also removes the occlusive effect of foreskin, such that micro-incontinence cannot lead to the pooling of urine and inflammation ^4^. The main indication for circumcision is LS-caused phimosis which has not responded to corticosteroid treatment ^2^; in fact, surgery may also reveal active disease on the glans or in the coronal sulcus, which later can be treated by corticosteroids ^2^. Circumcision has a cure rate of more than 90% in men with altered anatomy caused by scarring ^23^, although there are few data on long-term recurrence rates. Additionally, the biopsy taken during surgery may be used to confirm a clinical diagnosis of LS or facilitate the earlier detection of malignancy ^2^.

LS of the urethra is typically managed surgically, although Potts *et al*. ^37^ have found intraurethral steroids, applied onto a urinary catheter or meatal dilator, to be a safe and effective treatment for male LS patients with urethral strictures. Extended meatotomies often are performed in meatal stenosis or fossa navicularis and distal strictures to create a hypospadiac meatus ^38, 39^. In a cohort study of 16 patients with refractory fossa navicularis strictures, an extended meatotomy (first-stage Johanson manoeuvre) was found to be successful in 87% ^40^. Malone ^41^ described a different technique that combines dorsal and ventral meatotomies with an inverted V-shaped incision to avoid a hypospadiac meatus; however, no further studies have been performed on this technique.

Surgical reconstruction with urethroplasty, which involves either one or two stages, may be needed for severe complicated LS in the anterior urethra ( Table 2). Buccal mucosa is typically the graft of choice as it has a consistent and vascular lamina propria whilst mitigating the high risk of recurrence found with genital skin grafts ^39^. A one-stage repair is preferable when there is adequate residual urethral plate and preserved corpus spongiosum, dartos fascia and penile skin ^42^; it involves a dorsal onlay graft, where a dorsal urethrotomy is performed and the graft is secured to the corpora cavernosa; after providing treatment to 88 patients, Kulkarni *et al*. observed a 91% success rate over the course of 32.5 months of follow-up ^43^.

**Table 2.** Summary of types of urethroplasties performed in more complicated male lichen sclerosus cases.

Type of urethroplasty	Indications	Details of technique	Results
One-stage	Adequate urethral plate, corpus spongiosum, dartos fascia and skin ^42^	Dorsal urethrotomy Graft attachment to corpora cavernosa	91% success rate (32.5 months follow-up) ^43^
Two-in-one stage ^42^	No previous operation Navicular fossa involvement Adequate dartos fascia	Spongiofibrosis excision Create neo-urethral plate with buccal mucosa	90% success rate (16 months follow-up)
Two-stage ^38, 39^	Limited urethral plate	Graft attachment to tunica albuginea Maturation of graft Tubularisation of urethral plate	73 to 82% success rate
Perineal urethrostomy ^44, 45^	Recurrent urethral stricture disease or complex disease	Wide-based flap with an inverted U incision or the perineumbor using the 7-flap	93% success rate

In previously unoperated LS-related urethral strictures affecting the navicular fossa, a ‘two-in-one’ stage urethroplasty has shown a success rate of 90% at 16 months of follow-up. This technique involves the excision of the spongiofibrosis and the creation and tubularisation of a neo-urethral plate using buccal mucosa; it requires enough dartos to support the graft whilst allowing tissue mobility to reduce tension ^42^.

A two-stage penile urethroplasty is used to reduce the risk of recurrent disease or when the urethral plate is limited. The first stage involves the excision of the affected urethra via a midline penile incision and the opening of the glans before the buccal mucosa is grafted to the tunica albuginea and allowed to mature for 6 to 12 months. The second stage involves the incision and tubularisation of the urethral plate. Two series observed a success rate of 73 to 82%, although some patients elected not to undergo the second stage of repair ^38, 39^. Recurrences have been observed after both single- and two-stage repair techniques ^38^.

It is important to note that there is no gold standard for the surgical treatment of male LS. Reconstructive urologists often have differing opinions on the optimal surgical management of LS-induced urethral stricture disease because of, for example, differences in training and exposure to such patients ^46^.

Other methods of surgical management include glans resurfacing and skin grafting. Glans resurfacing is indicated in severe LS ^47^; it involves a circumcision and the removal of the penile glandular epithelium. A free split-thickness skin graft then can be harvested from the thigh and transplanted over the glans while using interrupted sutures throughout ^48^. Palminteri *et al*. showed a preference against using buccal mucosa as graft material as they observed some desquamation of the graft following exposure of air ^48^. All patients from one cohort study have reported subjective satisfaction in both aesthetic results and sexual function ^48^.

LS can present with concurrent buried penis, for which genital reconstruction may be required to restore sexual and urinary function and improve quality of life. The typical treatment for this condition involves an escutcheonectomy where the excess fat tissue, the escutcheon, between the waistline sulcus and inguinal creases is removed. Following a scrotoplasty, a split-thickness skin graft – from either the escutcheon or a thigh – can be used to reconstruct the penis. Patient satisfaction rates were observed to be greater than 80%, and the most common complication was local wound infection at a rate of up to 20% ^49^. A recent retrospective cohort study suggested that full-thickness skin grafts from escutcheon tissue could also be used with improved wound healing ^50^. Patient satisfaction was only reported subjectively, and 2 (15%) out of 13 had superficial wound infections. This technique avoids graft harvesting from an additional donor site. Although the long-term satisfaction and viability of this graft type have not been accessed, this technique remains promising ^50^.

## Future

LS is a debilitating disease. Future research should focus on elucidating the aetio-pathogenesis and true prevalence of LS so that the potentially devastating sequelae of poorly managed LS can be avoided. For example, the discovery of relevant epithelial susceptibility factors – by means of genotyping genomic DNA from peripheral blood – could give weight to the hypothesis that LS is caused by the chronic exposure of urine to occluded epithelium; understanding the aetiology of LS could give rise to earlier and more specific management options. There is a paucity of data on the management of LS; the long-term effects of the first-line treatment for LS, topical corticosteroids, are still relatively unknown, in particular for penile LS ^36^. Prospective multicentre studies could shed light on the efficacy of common management strategies such as circumcision or the process by which LS may progress to penile SCC. These suggestions are in line with the research questions posed by the recently published results of a Priority Setting Partnership ^51^.
